# Neoadjuvant chemotherapy with or without radiotherapy versus upfront surgery for resectable pancreatic adenocarcinoma: a meta-analysis of randomized clinical trials

**DOI:** 10.1016/j.esmoop.2022.100485

**Published:** 2022-05-14

**Authors:** I. Ghanem, D. Lora, N. Herradón, G. de Velasco, A. Carretero-González, M.Á. Jiménez-Varas, P. Vázquez de Parga, J. Feliu

**Affiliations:** 1 Department of Medical Oncology, Hospital Universitario La Paz (IdiPAZ), Madrid, Spain; 2 Clinical Research Unit (imas12-CIBERESP), Hospital Universitario 12 de Octubre, Madrid, Spain; 3 Department of Medical Oncology, Hospital Universitario 12 de Octubre, Madrid, Spain; 4 Helen Diller Family Comprehensive Cancer Center, University of California, San Francisco, San Francisco, USA; 5 Biblioteca Médica, Hospital Universitario La Paz, Madrid, Spain; 6 Department of Gastroenterology and Hepatology, Hospital Universitario Fundación Jiménez Díaz, Madrid, Spain; 7 Cátedra UAM-AMGEN, CIBERONC, Madrid, Spain

**Keywords:** resectable pancreatic cancer, neoadjuvant, upfront surgery, meta-analysis, randomized clinical trials

## Abstract

**Background:**

The role of neoadjuvant chemotherapy (NC) in resectable pancreatic cancer (RPC) has yet to be defined. This review aims to analyze the benefit of NC in RPC compared with upfront surgery (US) in terms of overall survival (OS) and disease-free survival (DFS).

**Patients and methods:**

PubMed, CENTRAL (The Cochrane Library), and Embase were systematically reviewed until 3 November 2021. Abstract proceedings and virtual meeting presentations from the American Society of Clinical Oncology and the European Society of Medical Oncology conferences, reference articles of published clinical trials, and review articles were considered. Only randomized clinical trials (RCTs) comparing NC administration with or without radiotherapy previous with surgery (experimental arm) versus US followed by adjuvant chemotherapy with or without radiotherapy (control arm) for RPC were included.

**Results:**

A total of 1135 studies were screened. Of these, 1117 studies were primarily excluded. Of the remaining 18 studies, 5 were excluded because of no adequate trial design for this work and 7 others had no available results. Finally, 6 trials with 469 patients with pancreatic cancer randomized to NC (*n* = 212) or US (*n* = 257) were selected. Compared with US, NC significantly improved OS [hazard ratio (HR) 0.75; 95% confidence interval (CI) 0.58-0.98; *P* = 0.033] and DFS (HR 0.73; 95% CI 0.59-0.89; *P* = 0.002). While the NC approach was not significantly associated with lower resection rate [relative risk (RR) 0.92; 95% CI 0.84-1.01; *P* = 0.069], the R0 resection rate was significantly higher for NC than for US (RR 1.31; 95% CI 1.13-1.52; *P* = 0.0004).

**Conclusion:**

This is the first meta-analysis of RCTs showing that NC improves OS for RPC compared with US followed by adjuvant therapy. Ongoing RCTs should confirm these findings with FOLFIRINOX to generalize the indication of NC.

## Highlights

• Nowadays, US followed by adjuvant treatment is the most accepted approach for RPC.
• NC improves DFS and OS in RPC.
• NC increases the R0 ReR in RPC.
• Ongoing randomized clinical trials should confirm the benefit of neoadjuvant FOLFIRINOX to generalize the indication.

## Introduction

The lower chance of cure for pancreatic cancer is widely known, reflected in the similarity of the mortality and incidence rates.^1^ Despite the relatively low incidence of pancreatic cancer compared with other tumors, its high lethality and increasing incidence have led to the estimation that pancreatic cancer is already the third cause of cancer death in the Unites States, after lung and colorectal cancer.^2^ Estimates also paint a similar picture for Europe by 2025.^3^

The nonspecific symptomatology at initial stages complicate the early diagnosis with only 15% of patients with potentially curable stage having resectable disease. Even in early stages the prognosis remains poor: in the hyper-selected good prognostic group of patients, limited to ‘resected resectable tumors’ able to receive adjuvant chemotherapy, the 3-year disease-free survival (DFS) rates are 24%-39%.^4^^,^^5^ The complicated location of the pancreas, surrounded by unresectable vascular structures easily infiltrated by the tumor, make the curative surgery a challenge. In addition, pancreatic cancer should be considered a systemic disease, given its ability to develop premature metastases even in the very early stages of the disease. Thus, the surgery, while essential, cannot be considered curative for 90% of the resected pancreatic adenocarcinomas.^6^ The development of new strategies to improve the results in this setting is therefore a priority.

For more than a decade, one of the most discussed approaches is the administration of neoadjuvant chemotherapy (NC) in the resectable stage. Among its multiple potential advantages, the early eradication of the micrometastatic disease seems to be the more pragmatic one. Furthermore, the higher chemotherapy completion rates compared with the adjuvant setting, the higher microscopic margin-negative resection rates (resection rates; R0 resection), or the selection of patients to undergo surgery after confirming the absence of progressive disease with chemotherapy favors its application. However, NC carries potential downsides such as the loss of the opportunity to carry out surgery due to progressive disease or chemotherapy-related clinical deterioration.

Based on the biological rational and some retrospectives studies and small prospective clinical trials, there remains an open question about the potential benefit of NC in resectable pancreatic cancer (RPC). Some international guidelines consider it an option in patients with poor prognostic characteristics.^7^ However, there is no high-level evidence to support it, and whether NC improves the outcome over adjuvant therapy in the resectable disease has not been definitively established.^8^

Several randomized clinical trials (RCTs) have tried to answer this question, but most have been prematurely closed because of a slow accrual.^9^, ^10^, ^11^, ^12^, ^13^ Other trials were designed with wide inclusion criteria for resectability, including borderline resectable disease.^13^^,^^14^ These limitations make it difficult to draw definitive conclusions by considering the studies individually.

Retrospective studies and meta-analyses suggest a benefit of neoadjuvant therapy in terms of overall survival (OS) and R0 resection rate.^15^, ^16^, ^17^ However, these meta-analyses were elaborated from retrospective or prospective nonrandomized studies, which once again limit the conclusions. One meta-analysis of RCT demonstrated the benefit of NC, although it also included studies evaluating borderline resectable disease.^18^

Recently, the results of various RCTs restricted to resectable disease have been communicated. This review aims to determine whether the administration of NC improves OS in RPC.

## Methods

### Study design and search strategy

We reviewed all RCTs comparing NC administration with or without radiation previous with surgery (experimental arm) versus upfront surgery (US) followed by adjuvant chemotherapy with or without radiation (control arm) for RPC. RPC was defined as no tumor contact with regional arteries (celiac artery, hepatic artery, and superior mesenteric artery), and regarding venous involvement, clinical trials with inclusion criteria of up to 180° of maximal venous contact were included. We excluded non-RCTs, observational, and retrospective studies. RCTs including resectable and borderline RPC were included only when data from the resectable disease cohort were specifically published and separately from borderline disease. When duplicated, only the most recent publication was included. The search strategy included multiple combinations of search terms and was limited to English language (Supplementary Table S1, available at https://doi.org/10.1016/j.esmoop.2022.100485).

To carry out the search, PubMed, CENTRAL (The Cochrane Library), and Embase were systematically reviewed until 3 November 2021. In addition, abstract proceedings and virtual meeting presentations from the American Society of Clinical Oncology and the European Society of Medical Oncology conferences, reference articles of published clinical trials, and review articles were considered. The article selection was carried out according to the Preferred Reporting Items for Systematic Reviews and Meta-Analyses (PRISMA) statement.^19^

### Outcomes, study selection, and data extraction

The main outcomes of the meta-analysis were OS and DFS and the secondary outcomes were resection rate and R0 resection rate. Two reviewers (IG and NH) independently applied the eligibility criteria for the selection of studies. In case of disagreement, both reviewers discussed and decided on inclusion of the study. Variables were extracted by one of these reviewers (IG) while the other (NH) checked the extracted information. Variables collected were first author’s surname, year of publication, National Clinical Trials (NCT) registry number, phase of the clinical trial, number of enrolled patients, number of arms, the treatment regimen in each arm, number of patients included in experimental arms for OS and for DFS, number of patients included in the control arms for OS and DFS, the median OS in experimental arms, the median OS in the control arms, the hazard ratio (HR) for the comparative OS arms with the confidence interval (CI), the median DFS in experimental arms, the median DFS in the control arms, the HR for the comparative DFS arms with the CI, the number of patients with tumor resection in each arm, and the number of patients with R0 in each arm.

### Statistical analysis and risk of publication bias

All statistical analyses were carried out in Stata (version 16.1; StataCorp, College Station, TX) using the meta command. The statistical heterogeneity assumption was evaluated by the χ^2^-based Cochran’s *Q* test (which was considered significant at a *P*-value of 0.05), and quantified with the *I*^2^ statistic (with values 25%, 25% to 75%, and 75% interpreted as representing low, moderate, and high levels of heterogeneity, respectively). HR with CIs were the parameters considered to assess the impact of treatment based on immune checkpoint inhibitors on OS and DFS as compared with standard of care. If HR and CI were not reported or the study had more than two arms,^20^ we applied indirect methods to obtain them.^21^ The relative risk (RR) was the effect measure for resection rate and R0 resection rate. The resection rate and R0 resection rate data from three trials were extracted from a pooled analysis recently published.^12^ Random effects models were used to pool studies and to correct the heterogeneity of the studies included. The funnel plot, which shows the relationship between the study standard error and effect size, was used to visually rejection publication bias.

### Risk of bias within the selected studies

We assessed the risk of bias including the randomization process, deviations from the intended interventions, missing outcome data, measurement of the outcome, and selection of the reported result. We assessed the risk of bias using the Cochrane Collaboration’s tool.^22^ Each item was evaluated as having low, high, or some concerns.

The protocol of this meta-analysis was published in PROSPERO with the registration number CRD42021247328

## Results

### Study selection and characteristics of the studies

**Figure 1:**
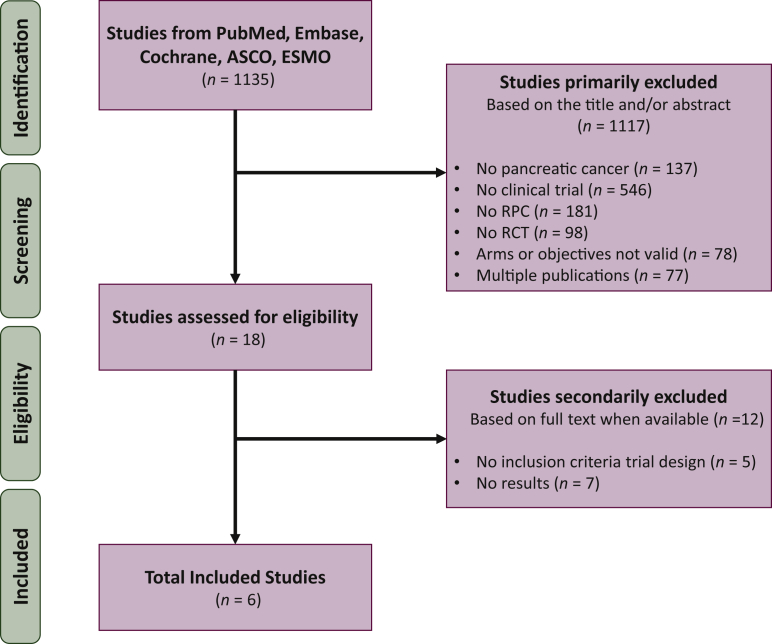
**Flow diagram of the selection strategy.** RCT, randomized clinical trial; RPC, resectable pancreatic cancer.

Our initial search strategy yielded 1135 studies (Figure 1). Of these, 1117 studies were primarily excluded. Among the remaining 18 studies, 5 were excluded because of no adequate trial design for this work and 7 others had no results (Supplementary Table S2, available at https://doi.org/10.1016/j.esmoop.2022.100485). Finally, six trials were selected for this meta-analysis, comprising 469 randomized patients with RPC (212 in the experimental NC arm and 257 in the control US arm).

### Special considerations about relevant excluded clinical trials

The nonpublished phase III Prep-02/JSAP05 study was excluded because patients with borderline resectable disease due to portal vein involvement were allowed to be included but no data for the resectable cohort were presented.^14^^,^^23^ The NEPAFOX study was also excluded from the analysis because patients with borderline RPC were included while the data were not limited to the resectable disease.^13^

### Special considerations about included clinical trials

Although the PREOPANC and Golcher et al. studies also included patients with borderline resectable neoplasms, data from the resectable disease cohort were published separately from borderline disease in both studies.^12^^,^^24^

All the studies included gemcitabine as neoadjuvant regimen (monotherapy or in combination with other drugs). The NC was administrated for at least 6 weeks in all trials. In three studies, radiation therapy was part of the neoadjuvant treatment in the experimental arm, while in another three the patients did not receive radiotherapy as neoadjuvant. In both the control and experimental arms, all trials included adjuvant gemcitabine-based chemotherapy after surgery. Four studies were prematurely closed, most of them because of poor recruitment.^10^, ^11^, ^12^^,^^20^ Table 1 shows the baseline characteristics of each clinical trial.

**Table 1:** Characteristics of clinical trials included in the meta-analysis

Study	Country	Phase	NC arm Neoadjuvant regimen (weeks)	NC arm Adjuvant chemotherapy (weeks)	US arm Adjuvant chemotherapy (weeks)	Sample size, *n*
Seufferlein et al.^26^	Germany	II	NabP-Gem (8)	NabP-Gem (16)	NabP-Gem (24)	118
Versteijne et al.^24^	The Netherlands	III	Gem (10) + RT	Gem (16)	Gem (24)	133^a^
Reni et al.^20^	Italy	II/III	PEXG (12)	PEXG (12)	1. Gem (24) 2. PEXG (24)	88
Birrer et al.^12^	Switzerland	III	Gem-Ox (8)	Gem (24)	Gem (24)	34
Casadei et al.^b^^,^^10^	Italy	II	Gem (12) + RT	Gem (24)	Gem (24)	38
Golcher et al.^b^^,^^11^	Germany	II	Cis-Gem (6) + RT	Gem (24)	Gem (24)	58^a^

Cis, cisplatin; Gem, gemcitabine; NabP, nab-paclitaxel; NC, neoadjuvant chemotherapy; Ox, oxaliplatin; PEXG, cisplatin, epirubicin, capecitabine, gemcitabine; RT, radiation therapy; US, upfront surgery.

a Patients with borderline resectable pancreatic cancer not included in the analysis.

b Actualized data from these studies were collected from Birrer et al.^12^

Regarding the resection margins, different criteria were adopted for defining R0 resection among the clinical trials included in this meta-analysis. For example, the PREOPANC or Casadei et al. trials considered the Royal College of Pathologist guidelines (R0: margin further than 1 mm distinct from any tumor cells), whereas the NEONAX study used the UICC classification (R0: no tumor cells within the resection margin).

### Overall survival and disease-free survival

**Figure 2:**
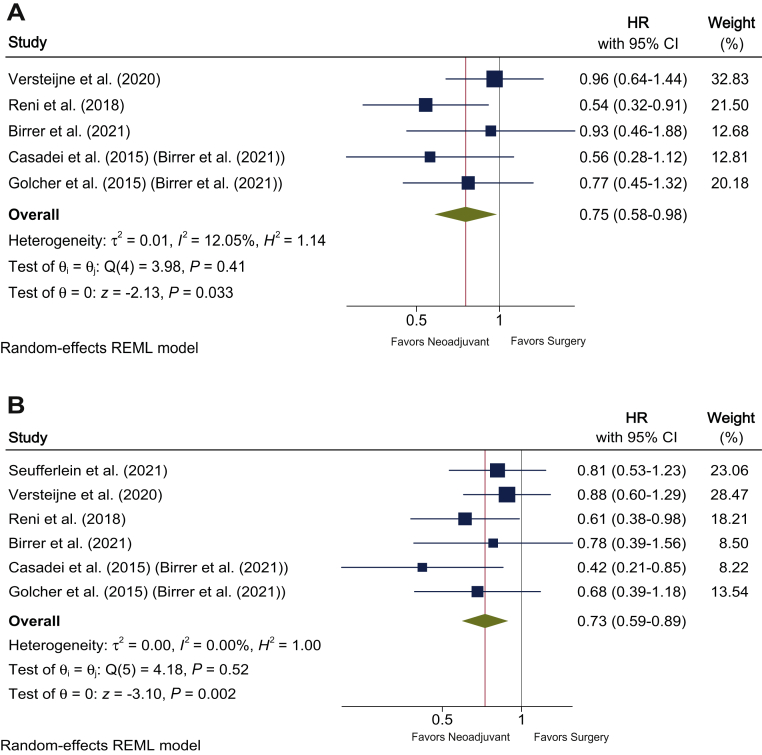
**Effect of neoadjuvant chemotherapy compared with upfront surgery on survival in resectable pancreatic cancer:** (A) overall survival; (B) disease-free survival. CI, confidence interval; HR, hazard ratio; REML, restricted maximum likelihood.

Four trials presented the HR for OS, one trial had the information for its estimation, and the last study had not communicated it at the time of our assessment.^10^, ^11^, ^12^, ^24^ The OS was significantly better for the neoadjuvant treatment strategy than for US (HR 0.75; 95% CI 0.58-0.98; *P* = 0.033). There was no significant heterogeneity (*I*^2^ = 12.05%; *P* = 0.41; Figure 2A). Four trials presented the HR for DFS and two trials had the information for its estimation. Neoadjuvant treatment significantly improved the DFS compared with US (HR 0.73; 95% CI 0.59-0.89; *P* = 0.002). There was no significant heterogeneity (*I*^2^ = 0.00%; *P* = 0.52; Figure 2B).

### Resection rate and R0 resection rate

**Figure 3:**
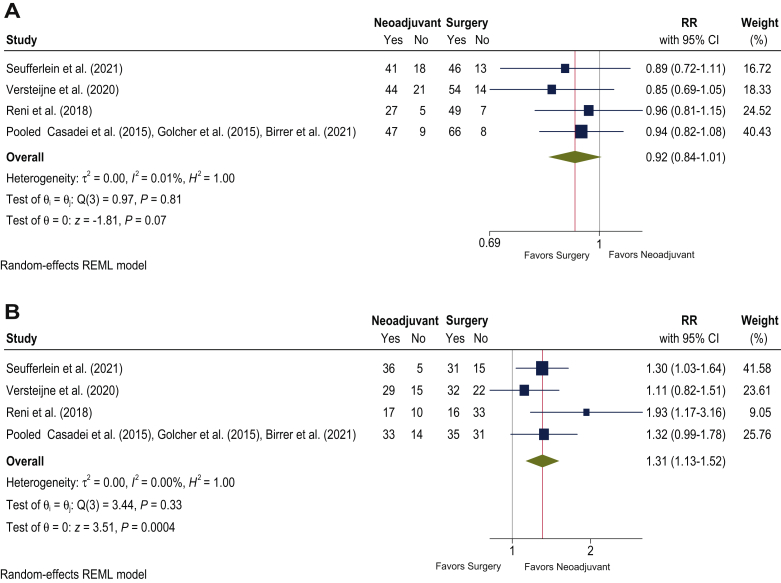
**Effect of neoadjuvant chemotherapy on resection in pancreatic cancer:** (A) relative risk (RR) of resection rates between neoadjuvant chemotherapy and upfront surgery; (B) RR of R0 resection rates between neoadjuvant chemotherapy and upfront surgery. CI, confidence interval.

Compared with US, the NC approach was not significantly associated with lower resesection rate (RR 0.92; 95% CI 0.84-1.01; *P* = 0.069; Figure 3A, Table 2). However, the R0 resection rate was significantly higher for the NC than for the US (RR 1.31; 95% CI 1.13-1.52; *P* = 0.0004; Figure 3B, Table 2). There was no significant heterogeneity for resection rate (*I*^2^ = 0.01%; *P* = 0.81) or R0 resection rate (*I*^2^ = 0.00%; *P* = 0.33).

**Table 2:** Resection rate and R0 resection rate for neoadjuvant chemotherapy (NC) and upfront surgery (US) approaches in each clinical trial

Study	Resection rate NC, %	Resection rate US, %	R0 resection rate NC, %	R0 resection rate US, %
Seufferlein et al.^26^	69	78	88	67
Versteijne et al.^24^	68	79	66	59
Reni et al.^20^	84	87	63	33
Birrer et al.^a^^,^^12^	84	89	70	53
Casadei et al.^a^^,^^10^				
Golcher et al.^a^^,^^11^				

a Data from these studies were updated and collected as a whole from Birrer et al.^12^

### Radiation therapy

Radiotherapy was part of the neoadjuvant treatment in three studies, including 229 patients (105 patients in the experimental arm and 124 in the control arm), whereas another three studies with 240 patients did not add radiotherapy to the NC (107 patients in the experimental arm and 133 patients in the control arm). There were no statistically significant differences in OS (*P* = 0.52) when comparing the addition of radiotherapy to the neoadjuvant approach (HR 0.82; 95% CI 0.61-1.10; *I*^2^ = 0.00%) with no addition (HR 0.67; CI 0.40-1.13; *I*^2^ = 32.63%) (Supplementary Figure S1A, available at https://doi.org/10.1016/j.esmoop.2022.100485). There were also no differences in DFS (*P* = 0.83) between administration of radiation therapy (HR 0.69; 95% CI 0.47-1.02; *I*^2^ = 39.50%) and no administration of radiation therapy (HR 0.73; 95% CI 0.54-0.97; *I*^2^ = 0.00%; Supplementary Figure S1B, available at https://doi.org/10.1016/j.esmoop.2022.100485).

### Risk of bias within the studies

Three studies were evaluated as having low risk of bias for the items analyzed. Another two studies were assessed as having some concerns for deviations from the intended interventions. The last study had some concerns regarding deviations from the intended interventions, missing outcome data, and selection of the reported results (Supplementary Figure S2, available at https://doi.org/10.1016/j.esmoop.2022.100485).

### Publication bias

Visual evaluation of the funnel plot did not show potential publication bias for OS, DFS, resection rate, or R0 resection rate (Supplementary Figure S3, available at https://doi.org/10.1016/j.esmoop.2022.100485).

## Discussion

This meta-analysis of RCTs for the first time has shown that there is an improvement in OS (HR 0.75) when patients with RPC receive NC compared with US. In addition, this meta-analysis has shown a relative increase in DFS with NC versus US (HR 0.73). Although a previously published meta-analysis did not show any improvement in OS,^25^ our study includes important updates, with the incorporation of two studies recently presented^26^ or published,^12^ the inclusion of the resectable cohort of a previous published trial lately analyzed^11^, ^12^ and the update of two studies,^10^, ^11^, ^12^ carrying out a separate analysis of the resectable cohort in previous published studies,^11^^,^^12^ and the update of two other,^12^ being the largest meta-analysis conducted in this context.

In early stage resected pancreatic cancer, adjuvant chemotherapy for 6 months is widely recommended because gemcitabine administration showed a benefit in OS compared with surgery alone.^6^ More recently, chemotherapy combinations such as capecitabine–gemcitabine, modified FOLFIRINOX or gemcitabine–nab-paclitaxel have shown variable OS benefits over gemcitabine alone.^4^^,^^27^^,^^28^

The OS in the US followed by adjuvant chemotherapy arms of the clinical trials included in this meta-analysis was much lower (16-26 months) than that achieved in contemporary adjuvant-only trials (where patients were included after tumor resection), both in experimental arms with the most active schedules (30-54 months) and in the gemcitabine alone arms (28-38 months).^4^^,^^27^^,^^28^ These large differences highlight the ‘hyper-selection’ of patients included in adjuvant-only clinical trials, where patients with occult metastases, discovered during or just after surgery, or patients with severe surgical complications or poor postsurgical recovery are excluded.

Our meta-analysis shows that the NC approach does not significantly decrease the resection rate (RR 0.92), while it does increase the R0 resection rate (RR 1.31). This fact can be interpreted as another clear advantage, because the administration of NC selects those patients who are most likely to benefit from surgery, thus avoiding the complications of unlikely curative R1/R2 resections. The R1 resections, defined as microscopic tumor within 1 mm of a resection margin, have a significantly poorer survival than R0.^29^ Despite the improvement of surgical results, up to 40% of patients experience complications after pancreatic resection with a postoperative mortality of ∼4%.^30^ In addition, the complications and/or clinical deterioration derived from surgery or the progressive disease can delay or avoid the administration of chemotherapy in patients with noncurative resections.

The administration of neoadjuvant radiation after chemotherapy could hypothetically improve the R0 resection rate. However, in the Alliance A021501 phase II RCT for borderline RPC, the mFOLFIRINOX plus hypofractionated radiation arm did not improve the OS compared with historical data with a median OS of 17.1 months and a R0 pancreatectomy rate of 56%.^31^ In the same trial, chemotherapy alone did present improvements over the historical data. In addition, the potential benefit from radiation for R0 resection at the resectable disease stage is inferior to the borderline RPC, and the role of radiotherapy as an adjuvant approach for local control remains at least controversial.^32^ This meta-analysis did not find differences for the addition of radiotherapy to the neoadjuvant approach in DFS or OS. However, the small number of studies and patients per group limits the drawing of definitive conclusions.

Another interesting point is whether, among patients with resectable disease, those with very early disease benefit less or not at all. The benefit of NC in the borderline resectable setting is highly relevant (HR for OS 0.61)^25^ and definitely established. The predefined subgroup analysis of the PREOPANC trial showed a benefit of neoadjuvant chemoradiotherapy for borderline RPC but not for RPC.^24^ However, in this trial, the inclusion criteria for considering resectable were no arterial involvement and venous involvement <90°, which are more restrictive than the National Comprehensive Cancer Network (NCCN) guidelines criteria (<180°). The NCCN guidelines consider the NC approach as an option only for patients with poor prognostics characteristics.^7^

Regarding the more appropriate chemotherapy regimen in this setting, it seems relevant to highlight that all the six RCTs used gemcitabine-based NC, two of them as monotherapy. Considering that (m)FOLFIRINOX has shown impressive benefits for OS in both the metastatic first-line and the adjuvant settings, (m)FOLFIRINOX could improve the results in the neoadjuvant setting as well. Interestingly, a meta-analysis found no difference between gemcitabine–nab-paclitaxel and FOLFIRINOX for cytoreduction.^33^ Nevertheless, a recently published phase II clinical trial that randomized 147 patients to receive perioperative (12 weeks preoperative and 12 weeks postoperative) mFOLFIRINOX or gemcitabine–nab-paclitaxel failed to improve the historical data from adjuvant, with a 2-year OS of 47% and 48% for mFOLFIRINOX and gemcitabine–nab-paclitaxel, respectively.^34^ In addition, the NEPAFOX trial, although without robust conclusions (stopped due to poor accrual), showed a numerically better mOS from adjuvant gemcitabine compared with neoadjuvant FOLFIRINOX for resectable and borderline resectable diseases. Besides, in none of the six clinical trials included in this meta-analysis the adjuvant chemotherapy in the control arm was mFOLFIRINOX, far superior to gemcitabine. Therefore, questions considering the best neoadjuvant regimen and the potential benefits of this approach compared with adjuvant (m)FOLFIRINOX remain unresolved. Several ongoing RCTs such as NorPACT-1, PANACHE01-PRODIGE48, PREOPANC-3, and Alliance A021806 are evaluating the neoadjuvant (m)FOLFIRINOX compared with US followed by chemotherapy and their results will help solve these issues.^35^, ^36^, ^37^, ^38^

At least 6 weeks of NC was administrated in all the included RCTs, reaching up to 12 weeks in two studies. Thus, when considering a neoadjuvant treatment, a minimum of 6-8 weeks must be recommended.

Precision medicine is another developing investigational area in pancreatic cancer. Up to 26% of patients with advanced pancreatic cancer harbor actionable molecular alterations with a likely benefit of targeted therapies.^39^ Thus, tumors with deficient mismatch repair may benefit from programmed death-ligand 1 inhibitors,^40^ whereas patients with germline *BRCA* or *PALB2* mutations show a benefit in PFS when treated with maintenance olaparib after platinum-based chemotherapy.^41^ The neurotrophic receptor tyrosine kinase (*NTRK*) gene fusion is a target for the treatment with larotrectinib or entrectinib. More recently, KRAS G12C inhibitors such as sotorasib or adagrasib have shown very promising results in early studies for patients with this mutation.^42^^,^^43^ Drugs against other classically nontargetable mutations such as *KRAS G12D,* present in 40% of pancreatic cancer cases, are currently under investigation.^44^ However, translating these findings to the early disease is still a long way off. In addition, the predictive factor of response to conventional therapies is being analyzed: DNA repair dysfunction is predictive of platinum sensitivity,^45^ while low GATA6 expression could identify resistance to FOLFIRINOX.^46^ These findings are under investigation in the neoadjuvant setting of the resectable disease.^47^

This study has several limitations: Most clinical trials were terminated due to poor accrual, with a low number of patients included and for a long period, which could lead to selection bias. Most of the studies were also underpowered or were not designed for a direct comparison. The studies have heterogenous neoadjuvant regimens with diverse duration of treatment, with some also adding radiation therapy to the neoadjuvant. In addition, the data from some studies are from subanalysis due to the inclusion of resectable as well as borderline resectable disease. Moreover, the definition for R0 resection was not uniform among the clinical trials included. However, this work has relevant strengths as well, such as being the first meta-analysis using data from only RCTs and strictly for RPC, including newly communicated clinical trials.

In conclusion, the neoadjuvant gemcitabine-based chemotherapy for RPC improves the DFS and OS compared with US followed by gemcitabine-based adjuvant therapy. The higher R0 resection rate and the early eradication of the micrometastatic disease can play a central role. Although the addition of radiation therapy as neoadjuvant can potentially increase the R0 resection rate, no conclusive results can be extracted from this analysis. Ongoing RCTs should confirm these findings and provide answers about the role of neoadjuvant (m)FOLFIRINOX at the resectable setting.

## Supplementary data

Supplementary Tables S1 and S2

Supplementary Figure S1 Effect of neoadjuvant chemotherapy with and without radiotherapy on survival in resectable pancreatic cancer. A. Overall survival. B. Disease-free survival; HR: Hazard ratio; CI: Confidence interval.

Supplementary Figure S2 Risk of Bias according to the Cochrane tool ROB2_IRPG_beta_v9.

Supplementary Figure S3 Funnel plots of A. Overall survival, B. Disease-free survival, C: Resection Rate, D. R0 Resection rate.
