# Supplementary material for: Neoadjuvant chemotherapy with or without radiotherapy versus upfront surgery for resectable pancreatic adenocarcinoma: a meta-analysis of randomized clinical trials
Source: ESMO Open. 2022 May 14;7(3):100485. doi: 10.1016/j.esmoop.2022.100485 (PMC9117867; doi:10.1016/j.esmoop.2022.100485)
Supplement: Supplementary Tables S1 and S2 [file mmc1.docx]

Supplementary materials

| Database | Search Strategy | Studies (N) |
| --- | --- | --- |
| Pubmed | (('surgery'/exp OR 'surgery':lnk OR surger*:ab,ti) AND (('pancreas tumor'/exp OR (('neoplasm'/exp OR neoplas*:ti,ab OR cancer*:ti,ab OR tumor*:ti,ab OR malignan*:ti,ab) AND ('pancreas'/exp OR pancrea*:ti,ab))) AND resectab*:ti,ab) AND ('neoadjuvant therapy'/exp OR ('neoadjuvant therapy':ti,ab OR 'neoadjuvant therapies':ti,ab OR 'neoadjuvant treatment':ti,ab OR 'neoadjuvant treatments':ti,ab OR 'neoadjuvant radiotherapy':ti,ab OR 'neoadjuvant radiation':ti,ab OR 'neoadjuvant systemic':ti,ab OR 'neoadjuvant chemotherapy':ti,ab OR 'neoadjuvant chemotherapies':ti,ab OR 'neoadjuvant chemoradiotherapy':ti,ab OR 'neoadjuvant chemoradiotherapies':ti,ab OR 'neoadjuvant chemoradiation':ti,ab))) AND (('randomized controlled trial'/exp OR 'randomized controlled trial' OR randomized) OR ('randomized controlled trial topic'/exp OR 'randomized controlled trial topic') OR ([controlled clinical trial]/lim OR [randomized controlled trial]/lim OR 'controlled clinical trial' OR 'randomized controlled trial')) | 237 |
| CENTRAL (Cochrane Library) | #1 MeSH descriptor: [Surgical Procedures, Operative] explode all trees  #2 ("surgery"):ti,ab,kw (Word variations have been searched)  #3 surger*  #4 #1 OR #2 OR #3  #5 MeSH descriptor: [Pancreatic Neoplasms] explode all trees  #6 MeSH descriptor: [Pancreas] explode all trees  #7 MeSH descriptor: [Neoplasms] explode all trees  #8 (pancrea*):ti,ab,kw (Word variations have been searched)  #9 (neoplas* OR tumor* OR cancer* OR malignan*):ti,ab,kw (Word variations have been searched)  #10 #6 OR #8  #11 #7 OR #9  #12 #10 AND #11  #13 #5 OR #12  #14 MeSH descriptor: [Neoadjuvant Therapy] explode all trees  #15 ("Neoadjuvant Therapy" OR "Neoadjuvant Therapies" OR "Neoadjuvant Treatment" OR "Neoadjuvant Treatments" OR "Neoadjuvant Radiotherapy" OR "Neoadjuvant Radiation" OR "Neoadjuvant Systemic" OR "Neoadjuvant Chemotherapy" OR "Neoadjuvant Chemotherapies" OR "Neoadjuvant Chemoradiotherapy" OR "Neoadjuvant Chemoradiotherapies" OR "Neoadjuvant Chemoradiation"):ti,ab,kw (Word variations have been searched)  #16 #14 OR #15  #17 #4 AND #13 AND #16 | 288 |
| EMBASE | ('surgery'/exp OR 'surgery':lnk OR surger*:ab,ti) AND ('pancreas tumor'/exp OR (('neoplasm'/exp OR neoplas*:ti,ab OR cancer*:ti,ab OR tumor*:ti,ab OR malignan*:ti,ab) AND ('pancreas'/exp OR pancrea*:ti,ab))) AND resectab*:ti,ab AND ('neoadjuvant therapy'/exp OR 'neoadjuvant therapy':ti,ab OR 'neoadjuvant therapies':ti,ab OR 'neoadjuvant treatment':ti,ab OR 'neoadjuvant treatments':ti,ab OR 'neoadjuvant radiotherapy':ti,ab OR 'neoadjuvant radiation':ti,ab OR 'neoadjuvant systemic':ti,ab OR 'neoadjuvant chemotherapy':ti,ab OR 'neoadjuvant chemotherapies':ti,ab OR 'neoadjuvant chemoradiotherapy':ti,ab OR 'neoadjuvant chemoradiotherapies':ti,ab OR 'neoadjuvant chemoradiation':ti,ab) AND ('randomized controlled trial'/exp OR randomized OR 'randomized controlled trial topic'/exp OR 'randomized controlled trial topic' OR [controlled clinical trial]/lim OR [randomized controlled trial]/lim OR 'controlled clinical trial' OR 'randomized controlled trial') | 435 |
| ASCO conferences | Resectable pancreatic cancer neoadjuvant clinical trial | 163 |
| ESMO congress (2017-2021) | Resectable AND pancreatic AND cancer AND Neoadjuvant AND clinical trial | 12 |
| **TOTAL** |  | **1135** |

**Table S1.** Search strategy

|  | Study | Exclusion reason |
| --- | --- | --- |
| 1 | Unno M, Motoi F, Matsuyama Y, Satoi S, Matsumoto I, Aosasa S, et al. Randomized phase II/III trial of neoadjuvant chemotherapy with gemcitabine and S-1 versus upfront surgery for resectable pancreatic cancer (Prep-02/JSAP-05). JCO. 2019 Feb 1;37(4_suppl):189–189. | Borderline resectable by portal vein allowed and data of the resectable cohort not available |
| 2 | Tachezy M, Gebauer F, Petersen C, Arnold D, Trepel M, Wegscheider K, et al. Sequential neoadjuvant chemoradiotherapy (CRT) followed by curative surgery vs. primary surgery alone for resectable, non-metastasized pancreatic adenocarcinoma: NEOPA- a randomized multicenter phase III study (NCT01900327, DRKS00003893, ISRCTN82191749). BMC Cancer. 2014 Jun 7;14:411. | Results not published or communicated |
| 3 | Labori K, Lassen K, Hoem D, Grønbech J, Søreide J, Mortensen K, et al. Neoadjuvant chemotherapy versus surgery first for resectable pancreatic cancer (Norwegian Pancreatic Cancer Trial - 1 (NorPACT-1)) - study protocol for a national multicentre randomized controlled trial. BMC surgery. 2017;17(1):94. | Results not published or communicated |
| 4 | Jang J, Han Y, Lee H, Kim S, Kwon W, Lee K, et al. Oncological Benefits of Neoadjuvant Chemoradiation With Gemcitabine Versus Upfront Surgery in Patients With Borderline Resectable Pancreatic Cancer: a Prospective, Randomized, Open-label, Multicenter Phase 2/3 Trial. Annals of surgery. 2018;268(2):215‐222. | Borderline resectable |
| 5 | Schwarz L, Vernerey D, Bachet J-B, Tuech J-J, Portales F, Michel P, et al. Resectable pancreatic adenocarcinoma neo-adjuvant FOLF(IRIN)OX-based chemotherapy - a multicenter, non-comparative, randomized, phase II trial (PANACHE01-PRODIGE48 study). BMC Cancer. 2018 Jul 24;18(1):762. | Results not published or communicated |
| 6 | Al-Batran S-E, Reichart A, Bankstahl US, Pauligk C, Kraus TW, Bechstein WO, et al. Randomized multicenter phase II/III study with adjuvant gemcitabine versus neoadjuvant/adjuvant FOLFIRINOX in resectable pancreatic cancer: The NEPAFOX trial. JCO. 2021 Jan 20;39(3_suppl):406–406. | Borderline resectable allowed and data of the resectable cohort not available |
| 7 | Testing the Use of the Usual Chemotherapy Before and After Surgery for Removable Pancreatic Cancer. ClinicalTrials.gov Identifier: NCT04340141. (Accessed on January 01, 2022). | Results not published or communicated |
| 8 | Neo-Adjuvant therapy or surgery in carcinoma pancreas CTRI/2019/05/019235 | Results not published or communicated |
| 9 | [Randomized phase II trial of neoadjuvant Chemotherapy with Gemcitabine and S-1 vs. without neoadjuvant chemotherapy in patients with resectable pancreatic cancer](https://www.cochranelibrary.com/central/doi/10.1002/central/CN-01855942/full) | Results not published or communicated |
| 10 | [Neoadjuvant chemoradiotherapy of GEM +S-1 for resectable pancreatic cancer : phase II study](https://www.cochranelibrary.com/central/doi/10.1002/central/CN-01903799/full) | Non-randomized clinical trial |
| 11 | Uggeri F, Caprotti R, De Grate L, Crippa S, Nobili C, Penati C, et al. Short-term preoperative IL-2 immunotherapy in operable pancreatic cancer: a randomized study. Hepato-gastroenterology. 2009;56(91‐92):861‐865. | No neoadjuvant chemotherapy at the experimental arm |
| 12 | Survival analysis after pre-surgical therapy in patients with resectable pancreatic cancer and risk factors | Results not published or communicated |

**Table S2.** Studies excluded after the eligibility process
