# Supplementary figures and images for: Neoadjuvant chemotherapy with or without radiotherapy versus upfront surgery for resectable pancreatic adenocarcinoma: a meta-analysis of randomized clinical trials
Source: ESMO Open. 2022 May 14;7(3):100485. doi: 10.1016/j.esmoop.2022.100485 (PMC9117867; doi:10.1016/j.esmoop.2022.100485)

A

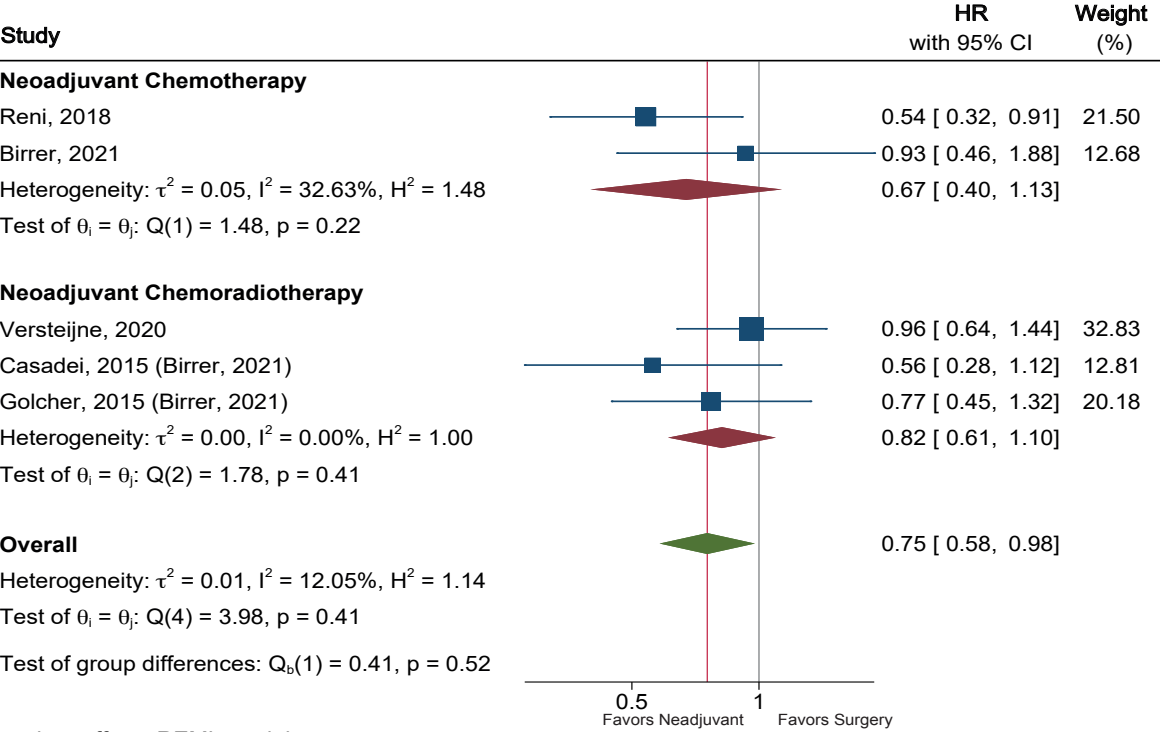

Random-effects REML model

B

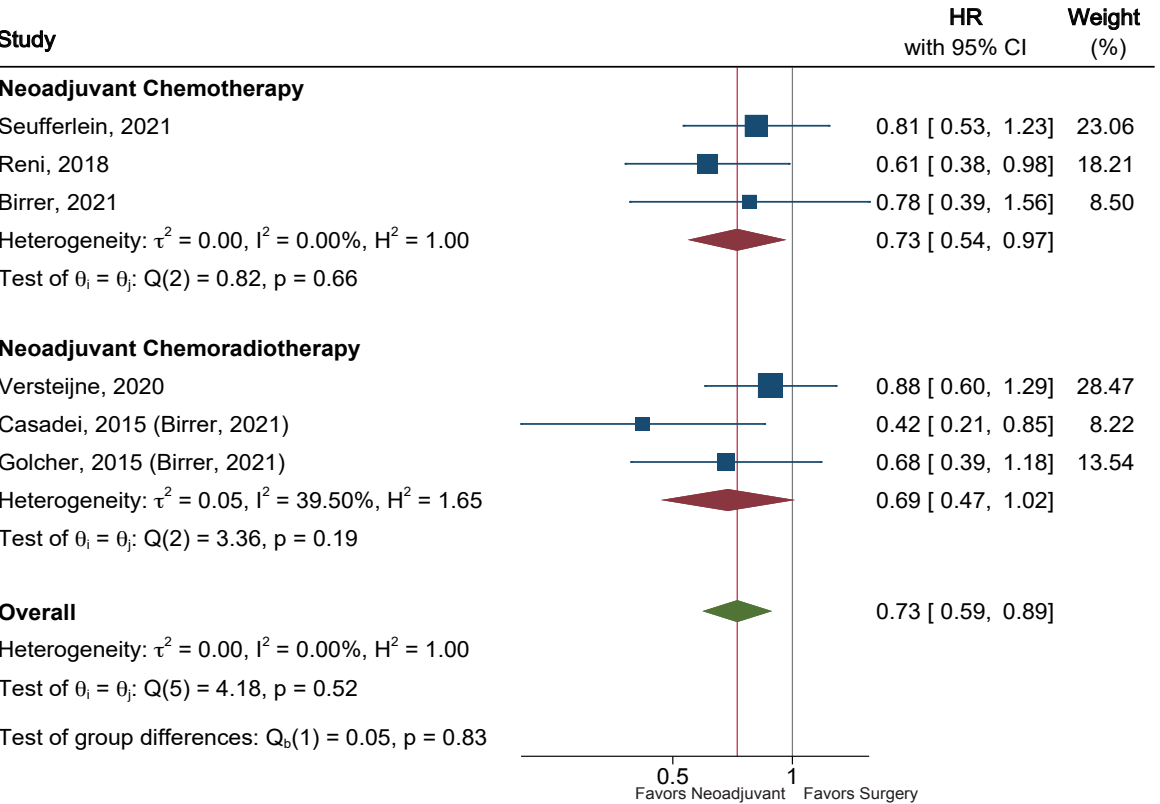

Random-effects REML model

Supplement: Supplementary Figure S1 — Effect of neoadjuvant chemotherapy with and without radiotherapy on survival in resectable pancreatic cancer. A. Overall survival. B. Disease-free survival; HR: Hazard ratio; CI: Confidence interval. [file mmc2.pdf]

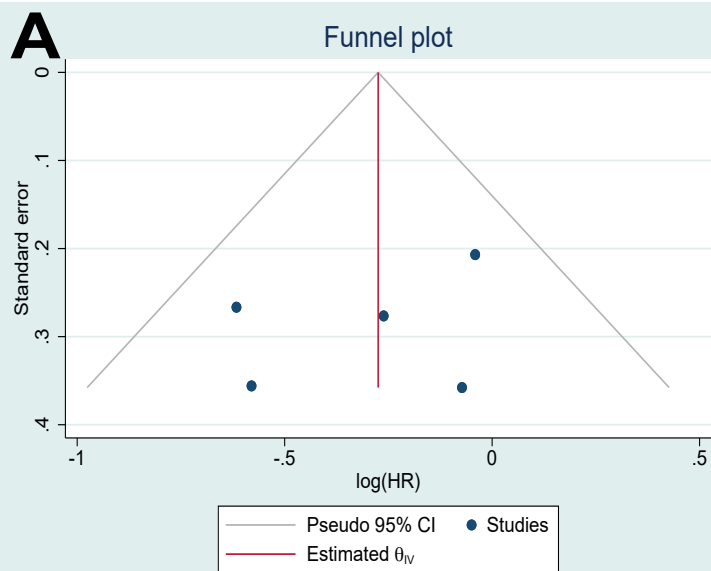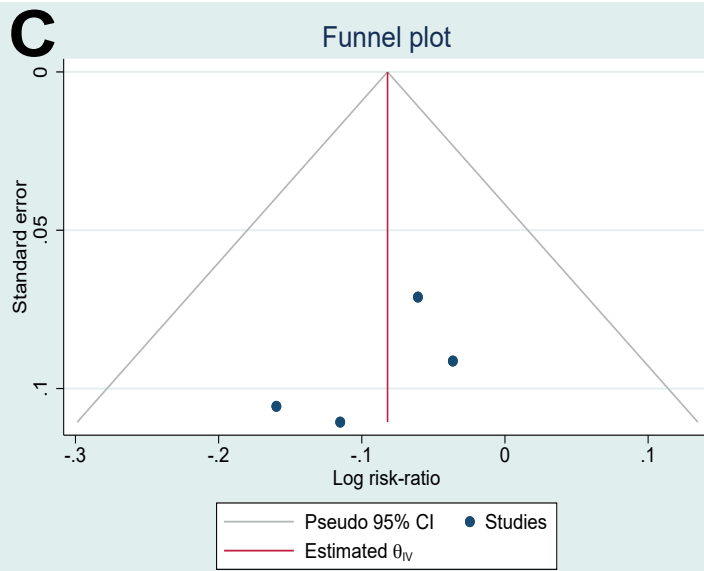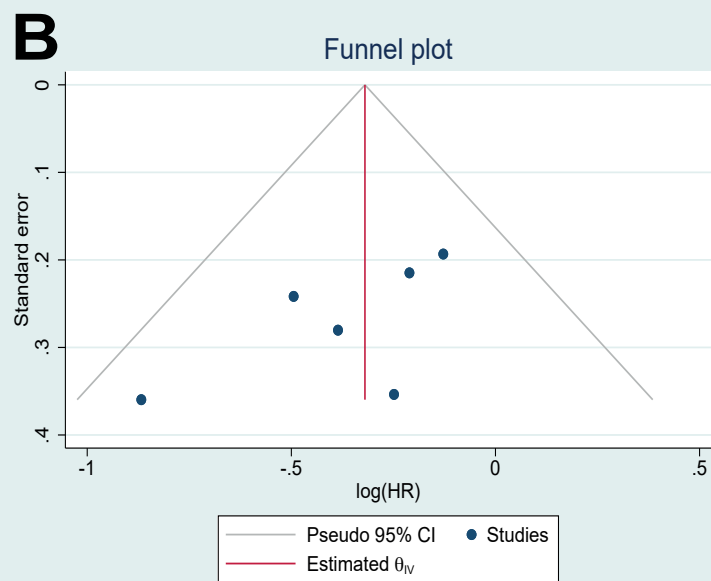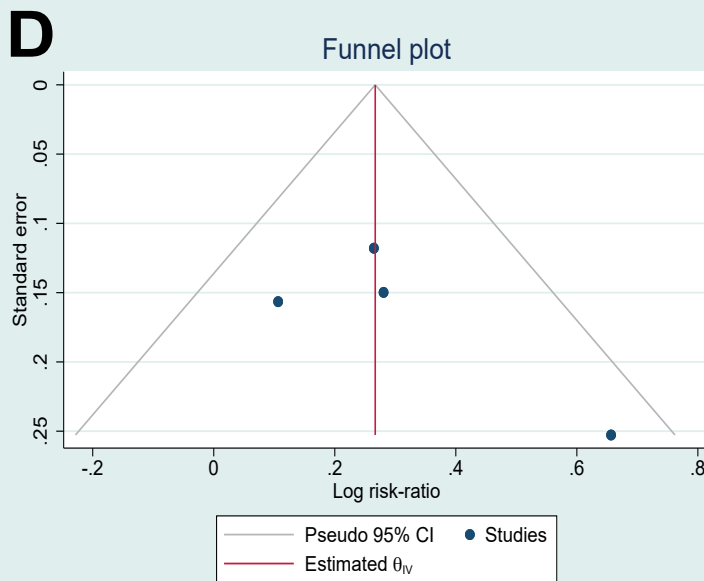

Supplement: Supplementary Figure S3 — Funnel plots of A. Overall survival, B. Disease-free survival, C: Resection Rate, D. R0 Resection rate. [file mmc4.pdf]
