# Supplementary material for: Neoadjuvant chemotherapy with or without radiotherapy versus upfront surgery for resectable pancreatic adenocarcinoma: a meta-analysis of randomized clinical trials
Source: ESMO Open. 2022 May 14;7(3):100485. doi: 10.1016/j.esmoop.2022.100485 (PMC9117867; doi:10.1016/j.esmoop.2022.100485)
Supplement: Supplementary Figure S2 — Risk of Bias according to the Cochrane tool ROB2_IRPG_beta_v9. [file mmc3.pdf]

	D1	D2	D3	D4	D5	Overall
Seufferlein, 2021	+	+	+	+	+	+
Versteijne, 2020	+	+	+	+	+	+
Reni, 2018	+	+	+	+	+	+
Birrer, 2021	+	!	+	+	+	!
Casadei, 2015 (Birrer, 2021)	+	!	+	+	+	!
Golcher, 2015 (Birrer, 2021)	+	!	!	+	!	!

+	Low risk
!	Some concerns
-	High risk
D1	Randomisation process
D2	Deviations from the intended interventions
D3	Missing outcome data
D4	Measurement of the outcome
D5	Selection of the reported result
